# Clinical outcomes of transforaminal endoscopic lateral recess decompression by using the visualized drilled foraminoplasty and visualized reamed foraminoplasty: a comparison study

**DOI:** 10.1186/s12891-020-03849-3

**Published:** 2020-12-10

**Authors:** Boyu Wu, Chengjie Xiong, Biwang Huang, Dongdong Zhao, Zhipeng Yao, Yawei Yao, Feng Xu, Hui Kang

**Affiliations:** 1 Orthopaedic Department, General Hospital of Central Theater Command of PLA, Wuhan, 430070 China; 2 The Second Clinical College of Chinese Medicine, Hunan University of Chinese Medicine, Changsha, 410208 China; 3 The First School of Clinical Medicine, Southern Medical University, Guangzhou, 51000 China

**Keywords:** Transforaminal endoscopic lateral recess decompression, Visualized drilled foraminoplasty, Visualized reamed foraminoplasty, Lateral recess stenosis, Disc herniation

## Abstract

**Background:**

Lateral recess stenosis (LRS) is a common degenerative disease in the elderly. Since the rise of comorbidity is associated with increasing age, transforaminal endoscopic lateral recess decompression (TE-LRD) is advocated. The objective of this study was to compare the clinical outcomes of TE-LRD in patients with LRS via visualized drilled foraminoplasty (VDF) or visualized reamed foraminoplasty (VRF) technique.

**Methods:**

A total of 45 and 42 consecutive patients with limp or unilateral radiculopathy symptoms underwent TE-LRD using the VDF and VRF technique, respectively. The radiation exposure and operation time, time to return to work, and complications were compared between two groups. Their clinical outcomes were evaluated with the visual analogue scale (VAS) leg pain score, VAS back pain score, Oswestry Disability Index (ODI) and modified MacNab’s criteria.

**Results:**

The average values of radiation exposure and operative time in the VDF group were significantly higher than those in the VRF group (*P* <  0.05). The postoperative VAS and ODI scores in both groups were significantly improved compared with those before the operation (*P* <  0.05). In addition, the VAS score of the leg pain and ODI score in the VRF group were significantly lower than those in the VDF group at the 1-week follow-up (*P* <  0.05). The good-to-excellent rates of the VDF group and VRF group were 88.89 and 90.48%, respectively, whereas the complication occurrence rates were 6.67 and 4.76% in the VDF group and VRF group, respectively.

**Conclusions:**

TE-LRD performed by using VRF technique can be applied to treat LRS safely and effectively with short radiation exposure and operation time. This technique was comparable to the VDF technique with improved VAS leg pain and ODI scores in the short period after the operation. However, potential complications and risks still need to be considered.

## Background

Lumbar spinal stenosis (LSS) is a common degenerative disease in the elderly and can be categorized into central stenosis, lateral recess stenosis (LRS) and foraminal stenosis [1, 2]. Surgery is indicated for patients with neurogenic claudication and radicular symptoms when conservative treatment has failed [3–5]. The pathogenesis of LRS is responsible for the compression of nerve roots caused by hypertrophic ligamentum flavum (LF) and facet joints with/without herniated intervertebral disc (IVD). The main purpose of surgical treatment is to decompress the spinal canal and relieve symptoms [6]. Compared with traditional open surgery, minimally invasive spine surgery (MISS) is associated with fewer postoperative complications, such as scarring, infection and restricted mobility [7]. Therefore, MISS is considered for patients with LSS if dynamic spinal instability wasn’t observed preoperatively [8, 9].

Percutaneous endoscopic lumbar discectomy (PELD) is one of the most applied MISS operations, and it is becoming increasingly popular in treating spinal degenerative disease. PELD appears to be a cost-effective procedure due to a short hospital stay, low postoperative costs of care and rapid rehabilitation with comparable clinical outcomes [10]. PELD was originally designed for IVD discectomy. Hoogland et al. reported that PELD under the transforaminal endoscopic spine system (TESSYS) was effective in treating IVD herniation with a success rate of 90% [11]. With the improvement of specialized instruments such as drill or reamer systems, the indication of this technique has expanded from lumbar disc herniation (LDH) to LRS. By using different instruments, the foraminal window can be widened to improve operative access and intracanal visualization. The lateral recess decompression via transforaminal endoscopic approach is termed as transforaminal endoscopic lateral recess decompression (TE-LRD) [12]. Shin et al. [13] have reported that adequate TE-LRD can be achieved by using reamed foraminoplasty technique with the aid of endoscopic drill system.

In addition to the endoscopic drill system, the endoscopic reamer system was also designed and developed. Visualized reamed foraminoplasty (VRF) technique under the endoscopy was developed from the traditional reamed foraminoplasty for the treatment of LRS [14]. The posterior wall of the lateral recess can be directly opened by dorsal bony plasty using this newly developed endoscopic reamer system. In this study, VRF and visualized drilled foraminoplasty (VDF) techniques were compared for the treatment of patients with LRS to analyze the safety and efficiency of the VRF technique.

## Methods

### Patient population

Between January 2016 and May 2018, 87 patients (51 males and 36 females), ranging from 43 to 80 years old (average 58.53 years), were enrolled in this study. The patients were categorized into two groups: VDF group or VRF group. The surgery was performed by two different senior surgeons. All the procedures of this study were approved by the institutional review board of General Hospital of Central Theater Command of PLA and were in accordance with the Helsinki Declaration. Written informed consent was obtained from each participant.

### Inclusion and exclusion criteria

The following inclusion criteria were used to select the patients: i) All participants complained of neurogenic claudication or unilateral radiculopathy symptoms; ii) Degenerative unilateral LRS localized at one segment was diagnosed on computed tomography (CT) scanning and magnetic resonance imaging (MRI), ventral compression was caused by disc herniation and osteophytes, dorsal compression was due to hypertrophic LF and facet, or both [13] (anteroposterior diameter of the lateral recess was < 4 mm) [15]; and the Bartynski Grading System was applied in our study [16]. iii) Neurological symptoms were consistent with CT scanning and MRI findings; iv) Conservative treatments failed to relieve the symptoms within 6 weeks. The following exclusion criteria were applied: i) Dynamic spinal instability was observed; ii) Central stenosis was combined with contralateral lateral recess stenosis at the same level; iii) Highly migrated discs; iv) Prior surgery at the same segment; v) Participants with severe systemic diseases who were unable to tolerate surgery. In the present study, all patients were informed objectively about the surgical procedure, benefits and potential risks, and each patient was able to freely select the surgical option.

### Surgical procedures

TE-LRD by using VDF Technique. Surgery was performed by using a transforaminal endoscopic spine system (Joimax®, Karlsruhe, Germany). Each patient underwent surgery in the prone position. The IVD space at the stenosis level was located using C-arm fluoroscopy. The distance between entry point and the midline was 12–16 cm. The entry point and approach angle were determined by preoperative imaging and intraoperative fluoroscopy. The procedure was performed as follows: 1) After local anesthesia, an 18-gauge needle was inserted into spinal canal at the entry point, and the guide wire was inserted after the core needle was removed; 2) A skin incision was made at the entry site for the dilator, and the dilator was passed over the guide wire until it reached the tip of superior articular process (SAP); 3) The foraminal window was widened by 4 graded reamer in sequence; 4) The final working cannula was introduced over the dilator and positioned properly; 5) An endoscope system was assembled by two irrigation channels and an eccentrically placed 2.7-mm working channel; 6) The foramen was widened vertically by using the high-speed drill under the endoscopy; 7) The posterior wall of lateral recess was undercut by high-speed drill, and then the hypertrophic LF was resected with Kerrison rongeur; 8) The herniated disc can be dissected by moving the working cannula to the disc if ventral neural compression was accompanied by dorsal neural compression; 9) Hemostasis was checked, and the endoscope was withdrawn after the decompression.

**Fig. 1:**
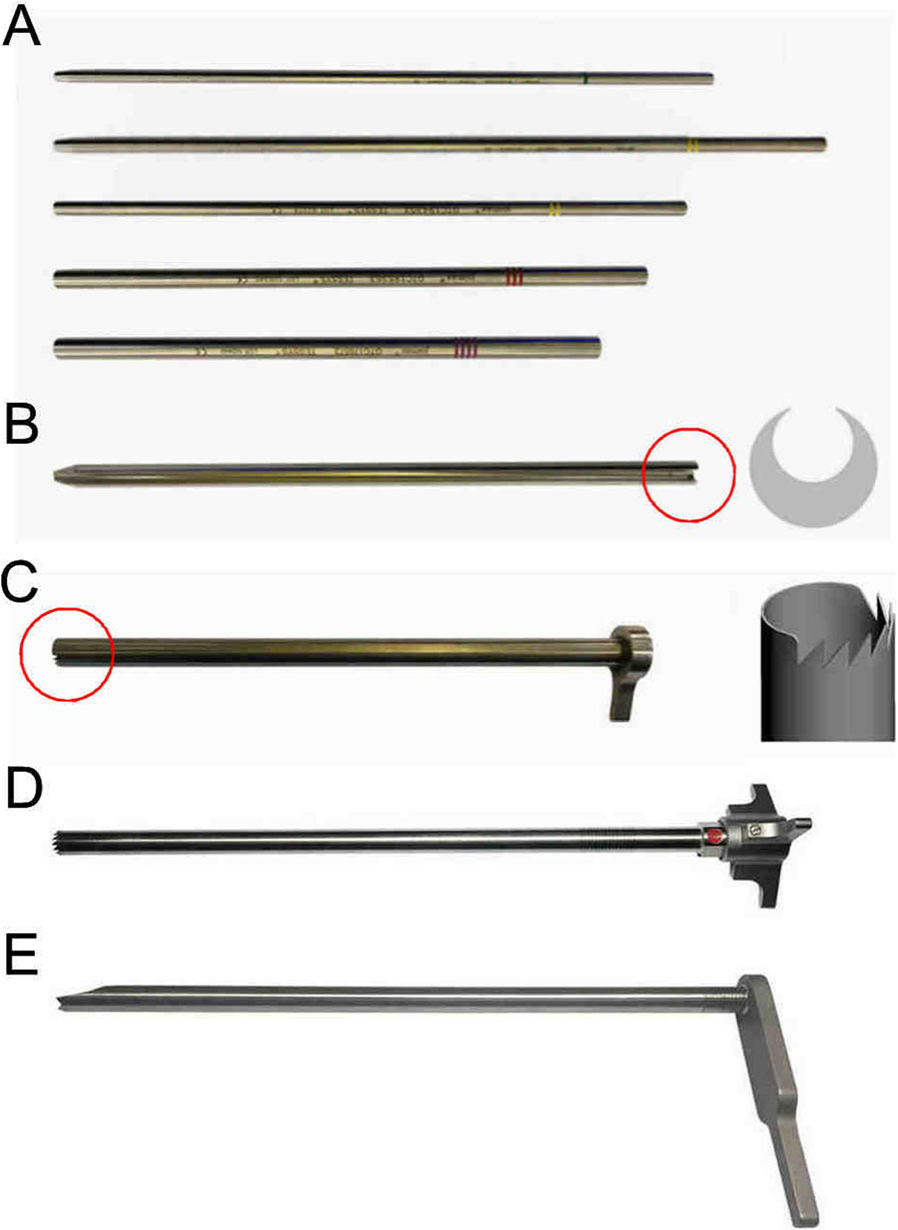
The instrumentation for visualized reamed foraminoplasty technique. **a** The sequential dilator and guide rod were applied to establish the portals once the 18-gauge needle was properly inserted. **b** The specially designed eccentric guide rod was inserted through the guide wire and secondary guide rod if the guide wire was not positioned at the target location, and we could change the direction of the guide wire by rotating the eccentric guide rod. **C** A half-serrated working cannula was inserted along the eccentric-shaft obturator for repositioning. **d** A special reamer was designed for the resection of osteophytes under the endoscopic system; **e** A sharp, bevel-ended working cannula was used for the decompression of the nerve roots

**Fig. 2:**
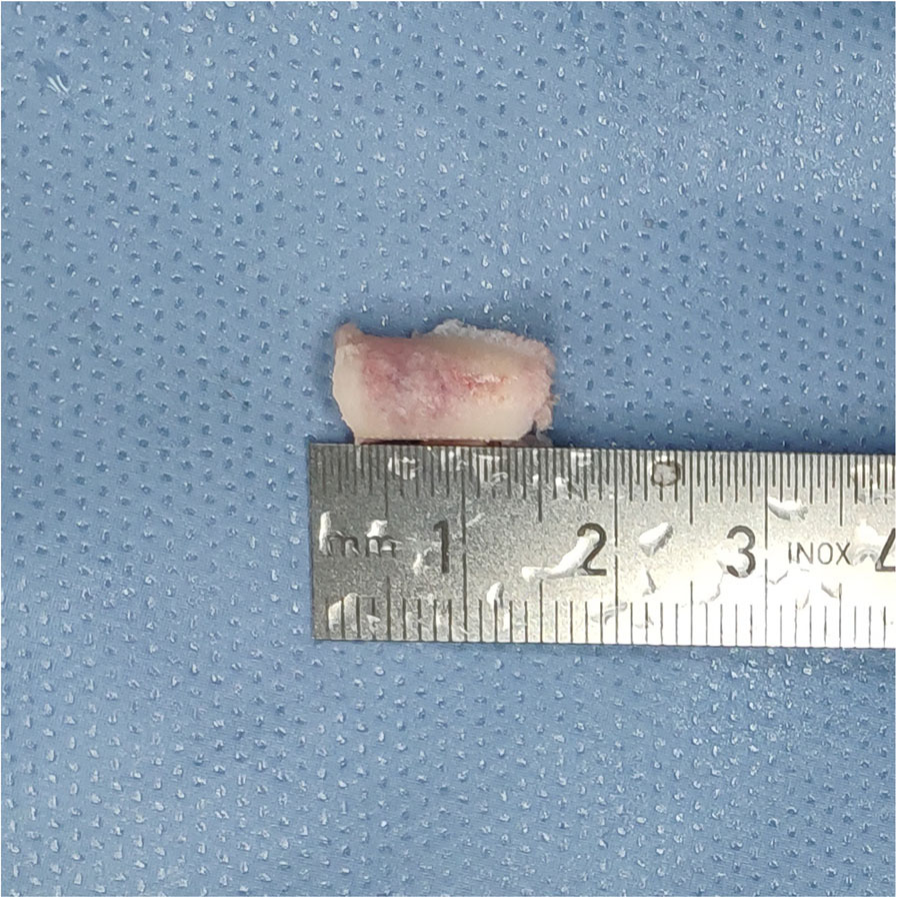
The bony plasty at the posterior wall of the lateral recess was performed by the reamer, and the “bone column” was removed

**Fig. 3:**
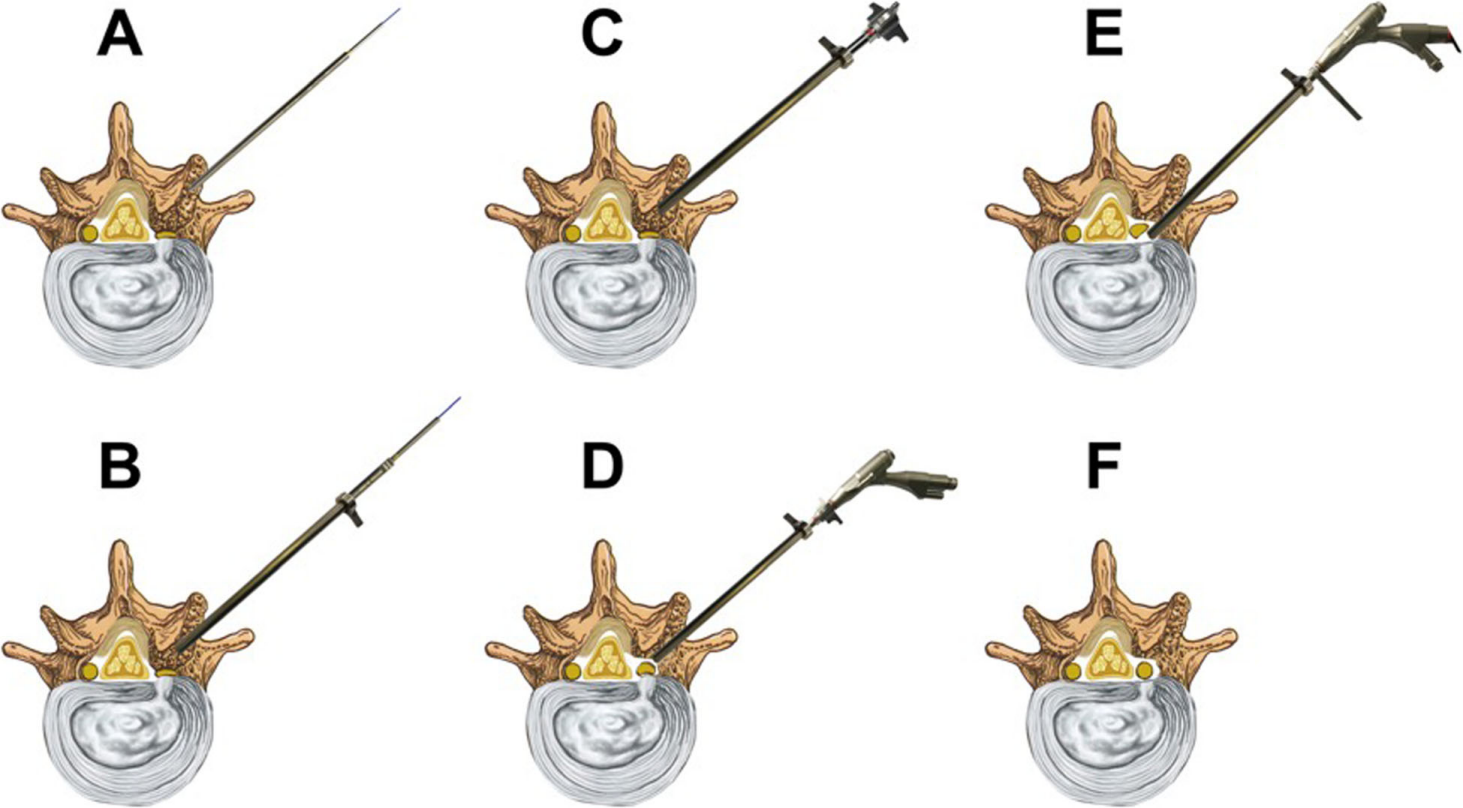
Illustration scheme of spinal canal decompression. **a** The dilator and guide rod were properly positioned. **b** The secondary guide rod and special eccentric guide rod were inserted through the guide wire step by step, and the half-serrated working cannula was inserted into the superior articular process along the eccentric guide rod. **c** The specially designed reamer was applied for the resection of osteophytes under the endoscopic system. **d** Dorsal decompression of the nerve roots; **e** ventral decompression of the nerve roots; **f** spinal canal decompression

TE-LRD by using VRF Technique. Surgery was performed using a transforaminal endoscopic spine system-ISEE (Joimax®, Karlsruhe, Germany). The instrumentation is shown in Fig. 1. All participants were in the prone position. The IVD space at the stenosis level was located using C-arm fluoroscopy. The distance between the entry point and the midline was 10–14 cm. The entry point and approach angle were determined by preoperative imaging and intraoperative fluoroscopy. The procedure was performed as follows: 1) After local anesthesia, an 18-gauge needle was inserted through the skin into the ventral portion of the SAP, and a skin incision was made at the entry site for guide wire; 2) The guide wire was introduced, and the needle was removed; 3) Sequential dilator (Fig. 1a) was inserted through the guide wire aiming at SAP; 4) Remove the dilator, and 2-grade dilator and specially designed eccentric guide rod (Fig. 1b) were inserted through the 2-grade dilator. Based on the fluoroscopic views of guide wire, rotating the eccentric guide rod around the 2-grade dilator to ideal targeting point. 5) A half-serrated working cannula (Fig. 1c) was passed over the eccentric guide rod to the base of SAP and positioned properly. 6) A specially designed reamer (Fig. 1d) and endoscope were introduced through the half-serrated working cannula (Fig. 1e). 7) Dorsal bony plasty at the posterior wall of the lateral recess was performed by the reamer, and the “bone column” was removed (Fig. 2). 8) Then, the hypertrophic LF was resected with Kerrison rongeur. 9) The half-serrated working cannula was replaced by a sharp, bevel-ended working cannula aiming at disc if ventral neural compression was present. 10) Under endoscopy, disc discectomy was performed until the nerve roots were decompressed. 11) Hemostasis was checked after decompression. The surgical procedure of VRF technique was illustrated in Fig. 3.

### Clinical assessment

The preoperative demographic data, including age, sex, body mass index (BMI), rate of diabetes and low extremity atherosclerosis disease, duration of symptoms, operative level, combined herniated disc, Bartynski grade, and follow-up time, were collected. We also recorded radiation exposure time, operative time, time to return to work, complication rate and recurrence. We evaluated each participant with a visual analogue scale (VAS) for back and leg pain and Oswestry disability index (ODI) questionnaires preoperatively and at each follow-up (1 week, 3 months and the latest follow-up). The VAS and ODI scores were recorded in the questionnaires at each follow-up in our outpatient clinic. The postoperative modified MacNab criteria [17] were also evaluated for clinical global outcome assessment at the latest follow-up. Sometimes, follow-ups were obtained by email or telephone communication.

### Statistical analysis

Statistical analysis was performed using SPSS 17.0 software (SPSS Inc., Chicago, USA). Measurement data were expressed as the mean ± standard deviation and were analyzed by student’s t-test. Enumeration data were analyzed by χ2 tests. *P* <  0.05 was considered to indicate a statistically significant difference.

## Results

### Comparison of basic demographic characteristics

Eighty-seven participants (VDF group, 45 cases; VRF group, 42 cases) who met the inclusion criteria were assessed in our study. The basic demographic characteristics (age, sex, BMI, comorbidities, duration of symptoms, operative level, combined herniated disc, bartynski grade and follow-up) were compared and presented in Table 1. There were no significant differences observed regarding the basic demographic characteristics between the two groups.

**Table 1:** Comparison of demographic characteristics in two groups

Characteristics	VDF group (*n* = 45)	VRF group (*n* = 42)	*P* value
Age (years)	59.18 ± 8.84	57.83 ± 7.54	0.45
Gender: (male) (%)	28 (62.22)	23 (54.76)	0.48
BMI (kg/m^2^)	25.10 ± 4.11	24.73 ± 4.18	0.68
Diabetes (%)	7 (15.56)	5 (11.90)	0.62
Low extremity atherosclerosis disease (%)	6 (13.33)	7 (16.67)	0.66
Duration of symptoms (months)	12.58 ± 6.12	14.12 ± 7.11	0.28
Operative level: (L4/5)/(L4/5 + L5/S1) (%)	31 (68.89)	26 (61.90)	0.49
With/without herniated disc	22 (48.89)	27 (64.29)	0.15
Bartynski grade: Bartynski grade 3/(Bartynski grade 2+ Bartynski grade 3) (%)	18 (40.00)	14 (33.33)	0.52
Follow-up (months)	23.29 ± 5.36	21.57 ± 5.10	0.13

The Age, BMI, Duration of symptoms, Follow-up were verified by student t-test; The Gender, Diabetes, low extremity atherosclerosis, operative level, combined herniated disc and Bartynski grade were verified by χ^2^ test. *P* < 0.05 represented significance. *VDF* visualized drilled foraminoplasty, *VRF* visualized reamed foraminoplasty

### Comparison of surgery-related indicators between the two groups

The intraoperative data showed that the average operative time (75.51 ± 15.63 min) and radiation exposure time (28.11 ± 7.13 s) in the VDF group were significantly higher than those (66.07 ± 11.23 min; 15.48 ± 5.01 s) in the VRF group (*P* < 0.05). However, there was no significant difference with regard to the time to return to work between the VDF group (12.02 ± 3.50 days) and the VRF group (10.95 ± 2.52 days) (Table 2).

**Table 2:** Comparison of surgery-related indicators between the two groups

Items	VDF group (*n* = 45)	VRF group (*n* = 42)	*P* value
Radiation exposure time(s)	28.11 ± 7.13	15.48 ± 5.01	**< 0.05**
Operation time (minutes)	75.51 ± 15.63	66.07 ± 11.23	**< 0.05**
Time to return to work (days)	12.02 ± 3.50	10.95 ± 2.52	0.11

The Radiation exposure time, operation time and time to return to work were verified by student t-test. *P* < 0.05 represented significance. *VDF* visualized drilled foraminoplasty, *VRF* visualized reamed foraminoplasty

### Comparison of clinical and functional outcomes

There was no significant difference between the two groups for the average VAS scores of back/leg pains and ODI scores at preoperation (*P* > 0.05); The average VAS scores of back/leg pains following surgery improved in both the VDF group and VRF group (*P* < 0.05). The average VAS score of back pain was reduced from 5.27 ± 1.12 to 1.47 ± 0.66 in the VDF group and from 5.05 ± 1.23 to 1.51 ± 0.57 in the VRF group. The average VAS score of leg pain was reduced from 7.36 ± 1.11 to 1.42 ± 0.71 in the VDF group and from 7.14 ± 1.03 to 1.48 ± 0.83 in the VRF group. In addition, the average ODI score following the operation also improved. The average ODI scores were reduced from 66.36 ± 9.87 to 21.02 ± 4.58 in the VDF group and from 69.52 ± 9.22 to 20.11 ± 5.49 in the VRF group (Table 3).

**Table 3:** Comparison of VAS and ODI scores in two groups

Items	VDF group (*n* = 45)	VRF group (*n* = 42)	*P* value
VAS of Back			
Preoperation	5.27 ± 1.12	5.05 ± 1.23	0.39
1-week after operation	2.31 ± 0.85	2.21 ± 0.75	0.58
3-month after operation	2.02 ± 0.81	2.07 ± 0.51	0.97
The latest follow-up	1.47 ± 0.66	1.51 ± 0.57	0.76
VAS of Leg			
Preoperation	7.36 ± 1.11	7.14 ± 1.03	0.36
1-week after operation	2.40 ± 0.72	1.95 ± 0.79	**< 0.05**
3-month after operation	1.93 ± 0.86	1.67 ± 0.72	0.12
The latest follow-up	1.42 ± 0.71	1.48 ± 0.83	0.66
ODI			
Preoperation	66.36 ± 9.87	69.52 ± 9.22	0.13
1-week after operation	34.80 ± 7.74	29.67 ± 5.91	**< 0.05**
3-month after operation	24.49 ± 5.61	22.81 ± 4.70	0.14
The latest follow-up	21.02 ± 4.58	20.11 ± 5.49	0.35

The VAS and ODI scores were compared by using student t-test. *P* < 0.05 represented statistical significance. *VDF* visualized drilled foraminoplasty, *VRF* visualized reamed foraminoplasty, *ODI* Oswestry dysfunction indexes, *VAS* visual analogue scale

**Fig. 4:**
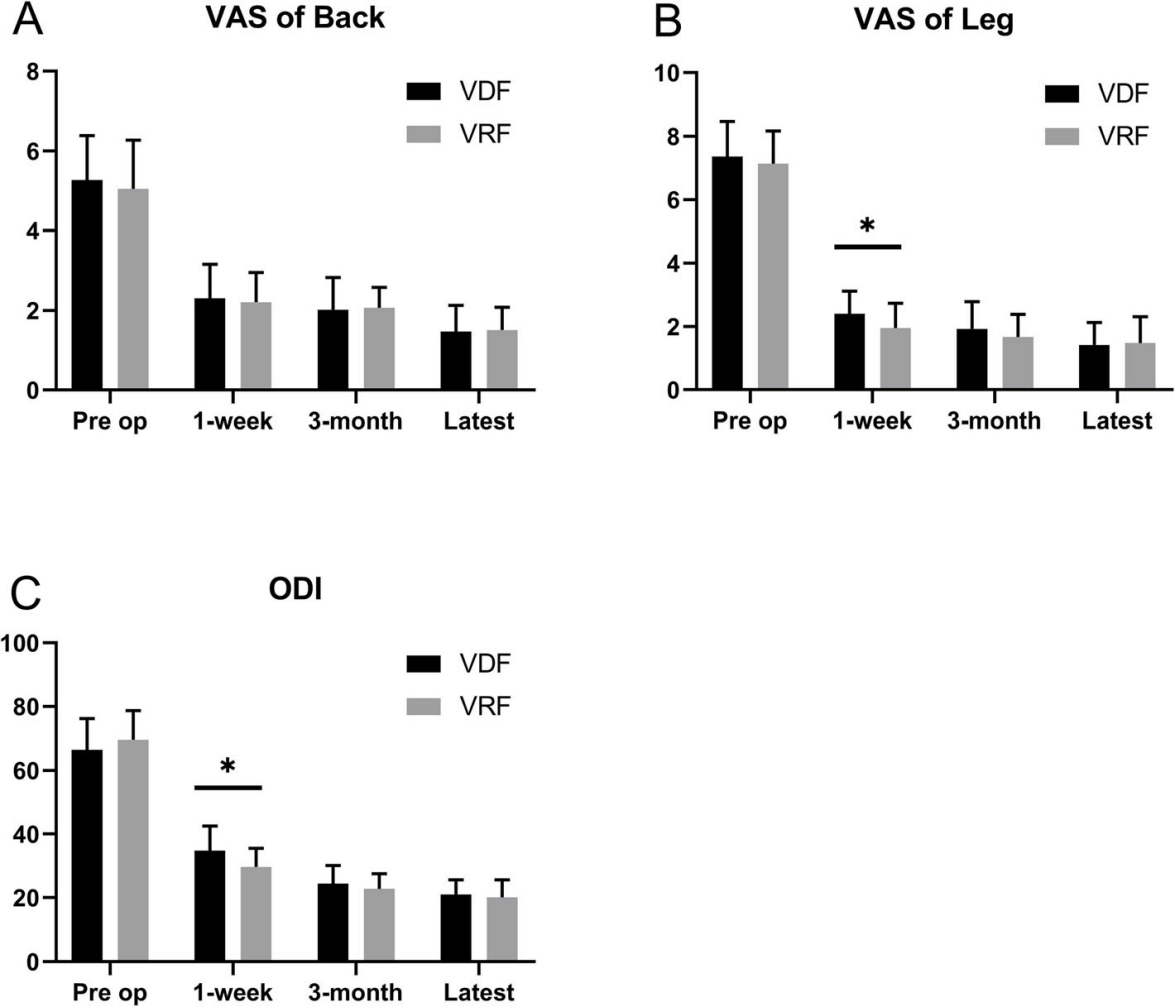
Comparison of VAS score of back (**a**), VAS score of leg (**b**) and ODI (**c**) at different time points. VAS, visual analogue scale; ODI, Oswestry Disability Index; VDF: visualized drilled foraminoplasty; VRF: visualized reamed foraminoplasty. * *P* < 0.05 VDF group vs. VRF group. Pre-op: Preoperation; 1-week: 1-week after operation; 3-month: 3-month after operation; Latest: The latest follow-up

There was also no significant difference between the two groups for the average VAS score of back pain (*P* > 0.05, Fig. 4a). Remarkably, the average VAS score of the leg pain in the VRF group was significantly reduced as compared with the VDF group at the 1-week follow-up; however, there was no significant difference between the two groups at the 3-month and the latest follow-up (*P* < 0.05, Fig. 4b). Similarly, the ODI score in the VRF group was significantly reduced as compared with that in the VDF group at the 1-week follow-up; however, there was no significant difference between the two groups at the 3-month and the latest follow-ups (*P* < 0.05, Fig. 4c).

Modified MacNab criteria were applied. The good-to-excellent rate in the VDF group was 88.89%, and the good-to-excellent rate in the VRF group was 90.48%. There was no significant difference in the good-to-excellent rate between the VDF group and the VRF group (*P* > 0.05, Table 4).

**Table 4:** Comparison of MacNab evaluation in two groups (n, %)

Groups	n	Excellent	Good	Fair	Poor
VDF group	45	19 (42.22)	21 (46.67)	4 (8.89)	1 (2.22)
VRF group	42	22 (52.38)	16 (38.10)	2 (4.76)	2 (4.76)
P value	0.62				

χ^2^ test was used to compare between each group. *P* < 0.05 represented significance. *VDF* visualized drilled foraminoplasty, *VRF* visualized reamed foraminoplasty

### Comparison of complications and recurrence

Complications occurred in three participants (6.67%) in the VDF group and two participants (4.76%) in the VRF group. Two participants in the VDF group experienced dysesthesia in the area distribution of the ipsilateral neighboring exiting nerve roots. Their symptoms recovered after physical treatment combined with medication. One participant in the VDF group complained of severe pain of the affected lower extremity, which was caused by nucleus pulposus omissions that were close to the traversing nerve roots. His symptoms vanished after the second surgical removal of the nucleus pulposus. Part of the facet joint of one participant in the VRF group was removed by the reamer; however, no spinal instability was observed postoperatively. One participant in the VRF group experienced a small sized dural tear and headache that recovered after medication and bed rest within 1 week. There were no severe complications such as vascular injury, cauda equina injury, abdominal content injury or surgical wound infection. There was no significant difference regarding to complication occurrence between the two groups (*P* > 0.05).

Only one participant in the VRF group with sciatica suffered from the same symptoms as preoperatively at 15 months after the operation. Participants with recurrent sciatica were subjected to transforaminal posterior lumbar interbody fusion (TLIF) when conservative management failed. The sciatica of this participant was relieved until the latest follow-up.

### Representative cases

**Fig. 5:**
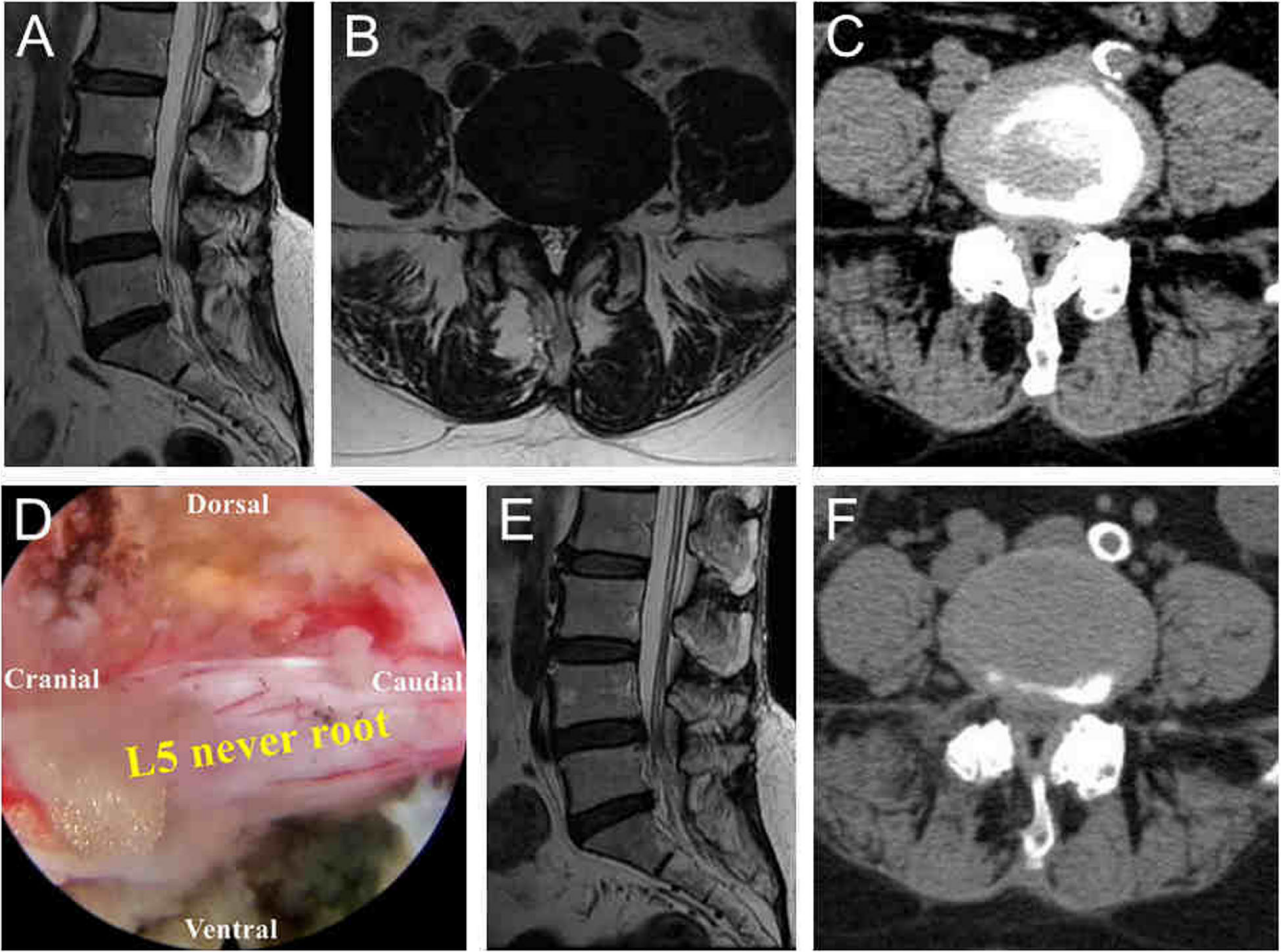
A case with lateral recess stenosis treated by the visualized reamed foraminoplasty technique. Preoperative MRI (**a**, **b**) and CT (**c**) of a 60-year-old woman with right leg radiating pain showed lateral recess stenosis and disc herniation of L4/5. The L5 nerve root was fully released after decompression (**d**). Postoperative MRI and CT showed that the lateral recess was enlarged, and the dorsal and ventral regions of the L5 nerve root were completely decompressed (**e**, **f**)

Representative cases who underwent TE-LRD via VRF technique are presented in Fig. 5.

## Discussion

This study retrospectively compared two different TE-LRD techniques (VDF and VRF) for the treatment of LRS. Although most of our participants experienced symptom relief following procedures, however, complications (5.75%) and recurrence (1.15%) still occurred. TE-LRD via VDF technique is emerging as an attractive minimally invasive surgical option in the treatment of LRS [13, 18, 19], and it is routinely applied to treat LRS at our institution. TE-LRD via VRF technique was newly designed to treat LRS [14]. However, no studies have been performed to compare this technique with previous minimal techniques. Therefore, VDR technique served as a reference to evaluate the efficiency and safety of this technique. These preliminary results demonstrated that TE-LRD via VRF technique is a feasible and safe way to treat LRS.

The full-endoscopic lumbar lateral recess decompression can be selectively applied according to the different types of LSS, including the interlaminar approach and transforaminal approach [2, 20]. The posterior interlaminar approach is suitable for central stenosis and LRS [2]. Rutten et al. [21, 22] reported that the clinical outcomes of full-endoscopic interlaminar operation were equal to those of open microsurgical decompression surgery. The lateral transforaminal approach is mainly used for the decompression of LRS and foraminal stenosis [2]. This lateral transforaminal approach is technically difficult due to the restricted field of vision and limited working mobility. Therefore, various specialized instruments have been developed to facilitate this surgical approach to overcome the anatomic limitations.

**Fig. 6:**
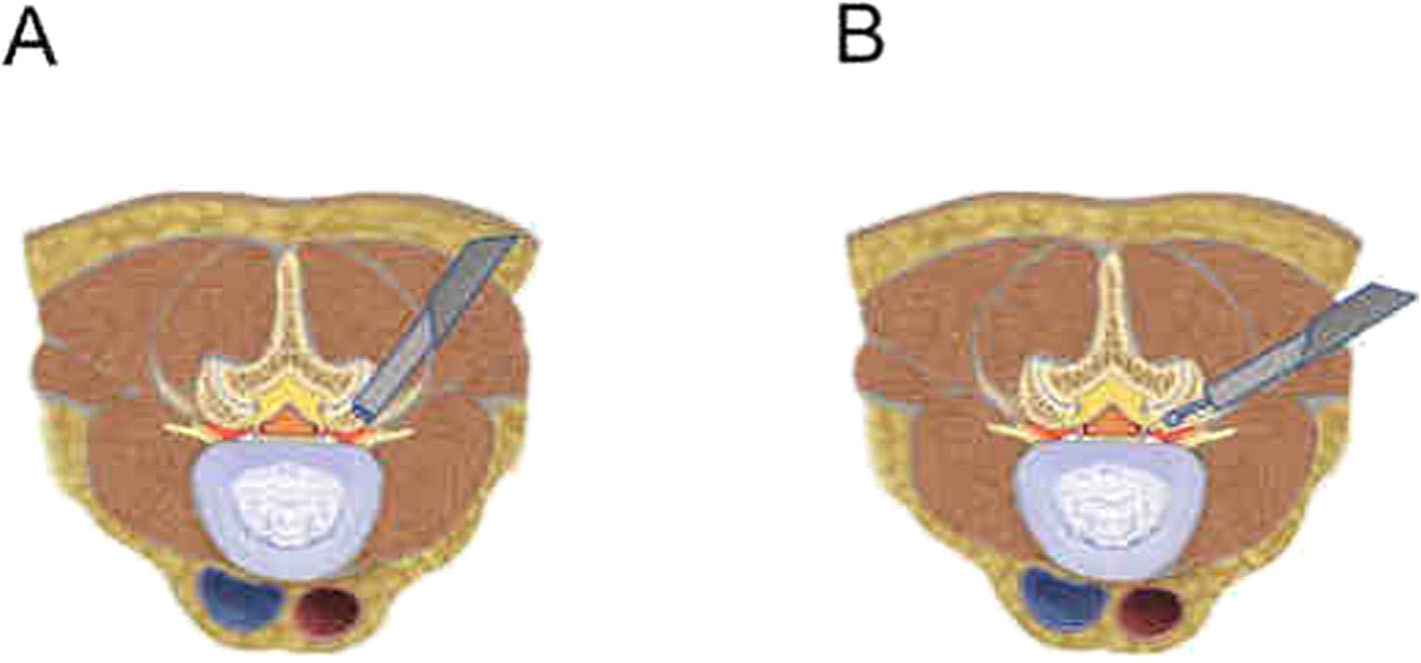
The comparison between visualized drilled foraminoplasty and visualized reamed foraminoplasty technique. **a** The bony plasty of the lateral recess by the endoscopic reamer. **b** The foraminoplasty by the endoscopic drill

Many studies have reported satisfactory results of patients with LRS following TE-LRD performed by using VDF technique [13, 23]. To achieve effective dorsal decompression of LRS, the distance from the midline to the skin entry point is farther than that of a typical transforaminal approach in these studies. This extreme lateral approach makes it possible to obtain good visibility when removing the LF. However, the potential risks of abdominal and vascular injury still should be cautioned by using this technique. Although such complications were not observed in our study, the identification of an appropriate trajectory before surgery is important for the prevention of this complication. Although the high-speed drill can also be applied to shape and sculpture the edges of articular osteophytes and expand the foramina precisely, it is time-consuming to acquire adequate space for surgical manipulation. Additionally, the high-speed drill may lead to exit nerve root injury, which is caused by thermal damage or vibration stimulation [24, 25]. VRF technique was developed from traditional reamed foraminoplasty technique; however, the principle of the VRF technique is different from that of typical reamed foraminoplasty technique. The puncture target of the VRF technique is the posterior element of nerve roots, so the distance from the midline to the skin entry point is shorter compared with a typical transforaminal approach. The site of foraminoplasty is at the base of SAP (In VDF group, the site of foraminoplasty is at the tip of SAP). If the guide wire is not positioned at the target location, we can change the direction by rotating the eccentric guide rod. After part of the SAP was removed by endoscopic reamer, the “bone-column” was removed for the exposure of LF. Therefore, there is enough working space left for us to perform dorsal decompression by resecting hypertrophic LF directly. By taking advantages of eccentric guide rods and endoscopic reamers, the radiation exposure time and average operative time in the VRF group were significantly shorter than those in the VDF group. Besides VRF technique, another reamer system has also been employed for the treatment of LRS. Li et al. [15] have reported that LRS can be treated by TE-LRD using a specially designed reamer. However, the reamer applied in this study was advanced with rotation under fluoroscopic guidance. This operation design is feasible; however, a steep learning curve must be overcome and potential risk of nerve root injury still exists despite such complication wasn’t reported in this study. Although the endoscopic reamer can ensure safe dorsal decompression in clear vision, two potential risks should be considered by using VRF technique. First, the endoscopic reamer might advance deeper in older osteoporosis patients due to inefficient control of the reamer handle. Second, this approach might affect the stability of the facet. Although no postoperative spinal instability was observed at the follow-ups until now, it still needs to be monitored during follow-up. The VDF and VRF techniques are compared in Fig. 6 and Table 5.

**Table 5:** Comparison between VDF and VRF Technique

Items	VDF	VRF
Approach	Transforaminal	Transforaminal
Puncture site	Paraspinal muscle 12–16 cm lateral to the midline	Paraspinal muscle 10–14 cm lateral to the midline
Puncture target	Tip of SAP	Ventral portion of SAP
Site of foraminoplasty	Tip of SAP	Base of SAP
Adjustment instrumentation of puncture	NO	Eccentric guide rod
Requirements for puncture accuracy	High	Low
Instrumentation of bony-plasty	Drill	Endoscopic reamer
Radiation exposure time	Long	Short
Operation time	Longer	Long
Facet disturbance	smaller	small
Lateral recess decompression	More precise	Precise

*VDF* visualized drilled foraminoplasty, *VRF* visualized reamed foraminoplasty

VAS scores for leg pain were significantly higher in the VDF group than those in the VRF group at the 1-week follow-up; however, there was no significant difference in leg pain between the two groups at the 3-month and latest follow-ups. These results might be due to the difference in the operation duration. A previous study has shown that a shorter operation duration is closely related to the reduced postoperative VAS in a short period [26]. There was no significant difference in VAS back pain between the two groups. Our participants also experienced less VAS back pain relief than VAS leg pain relief during the first week of follow-up. The removal of mechanical barriers, including the epidural fat and ligamentous structures, may exaggerate the tendency toward spinal instability, and spinal instability is an important cause of low back pain [27]. However, with the formation of granulation tissue and bone union, the spinal mechanical stability might be re-established, and symptoms were better relieved at later follow-ups. The ODI score is significantly correlated with VAS [28]. The difference in the VAS back and leg pain might be the explanation for the difference in the ODI. The decrease of 15 points in the ODI is perceived as effective [28], and the average decrease in the ODI at follow-ups was consistent with these criteria.

Dysesthesia is a common complication in patients treated with TE-LRD postoperatively. The incidence of dysesthesia following TE-LRD ranges from 2.3 to 5.4% [20, 25, 29]. The incidence of postoperative dysesthesia (4.44%) in the VDF group was consistent with previous findings, and no postoperative dysesthesia occurred in the VRF group. The possible reason for postoperative dysesthesia in the VDF group might be due to the stimulation exit nerve root caused by thermal damage or vibration stimulation of the drill. One participant in the VRF group experienced a small sized dural tear. Endoscopic reamer could advance deeper in patients with osteoporosis due to inefficient control of the reamer handle. Special caution should be paid to this risk. The recurrence that occurred in VRF group might be due to the following reasons: First, the participant was older than 60 years, and the elderly was at high risk for recurrent herniation after MISS operation [30]. Second, a non-recommended weight-bearing history was obtained postoperatively. In our study, the good-to-excellent rate in the VDF group was 88.89%, whereas the good-to-excellent rate in the VRF group was 90.48%. However, there was no significant difference with regard to the good-to-excellent rate between the two groups. These results are similar to previous studies [15, 31].

There were some limitations in the present study. First, this was a retrospective study with a small cohort of samples, and the follow-up was not long enough. A randomized, prospective and long-term follow-up study with a larger sample size is needed to test these findings. Second, each surgical procedure was performed by two different surgeons. The different skill levels of the two surgeons may also have an impact on the results. Ideally, all patients should be operated on by the same surgeon to minimize the impact of personal experience or skills on the results. However, we can’t get enough data from one surgical team for statistical comparison.

## Conclusion

TE-LRD via the VDF technique and VRF technique are both effective surgical options for the management of LRS. However, the VRF technique is shown to shorten radiation exposure and operation time and relieve VAS leg pain and ODI during the early period following the operation as compared with the VDF technique. The TE-LRD by using VRF technique for patients with LRS can be performed safely and effectively, and it might be regarded as a treatment alternative for LRS.

## Data Availability

The datasets for this study are available from the corresponding author upon reasonable request.

## Abbreviations

LSS: Lumbar spinal stenosis
LRS: Lateral recess stenosis
LDH: Lumbar disc herniation
LF: Ligamentum flavum
IVD: Intervertebral disc
MISS: Minimally invasive spine surgery
PELD: Percutaneous endoscopic lumbar discectomy
TE-LRD: Transforaminal endoscopic lateral recess decompression
VDF: Visualized drilled foraminoplasty
VRF: Visualized reamed foraminoplasty
CT: Computed tomography
MRI: Magnetic resonance imaging
SAP: Superior articular process
VAS: Visual analog scale
ODI: Oswestry disability index
